# Impact of Chronic Musculoskeletal Pain on Autonomic Function, Work Productivity, and Mood During Working Hours: A Pilot Cross-Sectional Study on IT Desk Workers

**DOI:** 10.7759/cureus.88089

**Published:** 2025-07-16

**Authors:** Yasumasa Oka, Takumi Jiroumaru, Yutaro Hyodo, Minoru Kuroda, Rina Murata, Takamitsu Fujikawa

**Affiliations:** 1 Department of Rehabilitation, Kanazawa Orthopedic and Sports Medicine Clinic, Shiga, JPN; 2 Department of Physical Therapy, Bukkyo University, Kyoto, JPN

**Keywords:** autonomic function, chronic musculoskeletal pain, during working hours, mood, work productivity

## Abstract

Objective

This study aimed to investigate the impact of chronic musculoskeletal pain (CMP), a prevalent issue among desk-based employees, on autonomic nervous function, work productivity, and mood during working hours.

Methods

The study enrolled 30 full-time software engineers employed at a Japanese information technology company. After excluding two participants because of incomplete heart rate variability (HRV) data, 28 individuals were included in the final analysis (15 with CMP and 13 without CMP). HRV was measured for 2.5 minutes between 15:00 and 16:00 using a photoplethysmographic accelerometer. Key indices included high-frequency power (HF), total power (TP), and the low-frequency to high-frequency ratio (LF/HF). Immediately following HRV assessment, participants completed the Two-Dimensional Mood Scale ([TDMS] subscales: vitality, stability, pleasure, arousal), the Single-Item Presenteeism Questionnaire (SPQ), and a CMP checklist. HRV parameters and TDMS scores were compared using independent-samples *t*-tests, while SPQ scores were analyzed using the Mann-Whitney U test. Effect sizes were calculated for all comparisons.

Results

There were no significant differences between groups for TP or LF/HF (*p* = 0.139 and 0.525, respectively). However, HF was significantly lower in the CMP group under a priori one-tailed testing (t(26) = −1.74, *p* = 0.047, Cohen’s *d *= 0.66), indicating diminished parasympathetic activity. SPQ scores were also significantly lower among participants with CMP (median: 80.0 vs. 92.0; U = 140.5, *p* = 0.042, *r* = 0.38). No significant group differences were observed across the four TDMS subscales (all *p* > 0.60, |*d*| < 0.20).

Conclusions

Desk workers with CMP exhibited reduced parasympathetic function and self-reported work productivity despite reporting comparable momentary mood states. These findings suggest that the combined use of HF-HRV and the single-item SPQ may offer a feasible approach for screening and monitoring workplace pain-related risk. Further longitudinal and interventional studies are warranted to establish causal relationships.

## Introduction

Chronic pain affects an estimated 10-30% of the Japanese population, with low back, neck, and shoulder pain representing the most frequently reported complaints [1-3]. Among these conditions, chronic musculoskeletal pain (CMP) has emerged as a key contributor to diminished occupational productivity, posing a significant socioeconomic burden for both employees and employers [4,5]. Productivity loss attributable to health issues is typically categorized into absenteeism (absence from work) and presenteeism (reduced performance while at work despite physical presence) [6]. Notably, the economic cost of presenteeism has been estimated to be approximately threefold higher than that of absenteeism [7], underscoring the urgent need for effective interventions to mitigate CMP in workplace settings. Importantly, a growing body of evidence indicates that chronic pain is frequently associated with autonomic dysfunction, including imbalances in sympathetic and parasympathetic nervous system activity [8,9].

Heart rate variability (HRV) is a well-established, non-invasive physiological indicator of autonomic nervous system activity [10,11]. Prior studies have demonstrated that individuals with CMP commonly exhibit reduced parasympathetic tone, reflected by decreased high-frequency power (HF), a marker associated with chronic stress and autonomic imbalance [12]. Although the association between HRV and CMP has been investigated under laboratory conditions and during routine daily activities, limited research has explored this relationship in real-time during actual working hours.

The Two-Dimensional Mood Scale (TDMS) is a brief, validated self-report instrument that evaluates four transient mood states - vitality, stability, pleasure, and arousal - within seconds [13]. Despite its ease of use, its relationship with CMP in occupational settings has yet to be elucidated. Additionally, the Single-Item Presenteeism Questionnaire (SPQ), developed at the University of Tokyo, measures self-reported productivity loss over the past four weeks as a percentage [14]. Its brevity and practicality make it a promising tool for assessing the functional impact of CMP in office environments.

In this context, the present pilot cross-sectional study aimed to examine whether CMP is associated with (i) reduced parasympathetic activity, as indicated by HF-HRV, and (ii) decreased self-reported work productivity, as measured by the SPQ, among desk-based workers in a Japanese information technology (IT) company. As an exploratory aim, we also assessed differences in the four TDMS mood indices between individuals with and without CMP. We hypothesized that the CMP group would exhibit lower HF and SPQ scores and that the pattern of TDMS subscale scores would differ from that of the non-CMP group.

By concurrently evaluating physiological, psychological, and productivity-related metrics during working hours, this study seeks to provide preliminary evidence to inform the design of future large-scale longitudinal and interventional investigations targeting workplace pain management.

## Materials and methods

Study design

This cross-sectional pilot study was conducted among desk-based employees working in the development division of an IT firm. Data collection was carried out over a two-month period (September to October 2024). All assessments were performed during standard working hours, specifically between 15:00 and 16:00, to minimize potential circadian influences on physiological measurements.

Participants

The target division comprised approximately 80 full-time software engineers. Human resources personnel distributed a study invitation via email to employees who met the inclusion criteria and none of the exclusion criteria. Eligibility criteria included (1) full-time employment within the division, (2) age between 20 and 60 years, and (3) provision of written informed consent. Exclusion criteria were (1) the presence of serious medical conditions that could interfere with study participation (e.g., fractures, severe musculoskeletal disorders, malignancies), (2) a diagnosis of cardiac disease or pacemaker implantation, and (3) current treatment for psychiatric illness. A total of 30 employees consented to participate, with two being excluded owing to incomplete HRV data, resulting in a final analytical sample of 28 participants (23 men, 5 women; mean ± SD: 40.2 ± 12.6 years). Among them, 15 individuals met the criteria for CMP, defined as low back, neck, or shoulder pain persisting for ≥3 months and occurring ≥2 days per week over the past year. The remaining 13 participants reported no such symptoms.

Procedure and measures

Heart Rate Variability

HRV was assessed using a finger-mounted photoplethysmographic accelerometer (Pulse Analyzer Plus View, TAS9View; YKC Inc., Tokyo, Japan). The device’s validity and reliability have been previously demonstrated [15]. Participants were instructed to abstain from vigorous physical activity and caffeine intake for at least 2 hours prior to testing. After a 5-minute seated rest period, a single 2.5-minute HRV recording was obtained. Frequency-domain indices were computed using Kubios software, including total power (TP), HF (0.15-0.40 Hz, an index of parasympathetic activity), and the low-frequency to high-frequency ratio (LF/HF) reflecting sympathovagal balance [16].

Mood

Following HRV measurement, participants completed the short form of the Two-Dimensional Mood Scale (TDMS-ST) [13,17]. This self-report instrument provides four subscale scores - vitality, stability, pleasure, and arousal - each rated on a 6-point Likert scale. Permission to use the TDMS-ST, which is not an open-access instrument, was purchased from IMF Co., Ltd. (Tokyo, Japan) and formally granted by the developer (Y. Sakairi, personal communication).

Presenteeism

Self-reported work productivity was assessed using the SPQ developed at the University of Tokyo [14]. Participants rated their performance over the preceding four weeks as a percentage of their best possible performance (0-100%). Presenteeism loss was calculated as 100% minus the SPQ score. The single-item SPQ is in the public domain for non-commercial research and therefore required no additional licensing.

Chronic Pain

CMP status was classified as present or absent based on responses to a standardized checklist addressing musculoskeletal pain in the neck, shoulders, and low back, in accordance with national guidelines [2,18].

Data analysis

All data were anonymized prior to analysis. Participants were stratified into two groups based on CMP status: CMP-positive (CMP+) and CMP-negative (CMP-). The distribution of continuous variables was assessed using the Shapiro-Wilk test. TP, HF, LF/HF, and TDMS subscores were normally distributed (p > 0.05) and therefore analyzed using independent-samples t-tests. In contrast, the SPQ data were non-normally distributed (p < 0.05) and compared using the Mann-Whitney U test. Effect sizes were calculated and reported as Cohen’s d (or Hedges’ g in cases of small sample size) for parametric tests and as r for non-parametric comparisons. Statistical significance was defined as α = 0.05 (two-tailed). All analyses were performed using SPSS Statistics version 28 (IBM Corp., Armonk, NY).

Ethical considerations

This study was conducted in accordance with the ethical principles outlined in the Declaration of Helsinki and received approval from the Kanazawa Orthopedic Clinic Ethics Committee (Approval No. Kanazawa-OSMC-2024-004). Written informed consent was obtained from all participants, who were informed of their right to withdraw from the study at any time. All data were stored in a de-identified format and utilized exclusively for academic research purposes.

## Results

A total of 28 participants were included in the final analysis: 15 with chronic musculoskeletal pain (CMP+; 13 men, 2 women; mean age ± SD 43.8 ± 13.3 years) and 13 without CMP (CMP−; 10 men, 3 women; 37.9 ± 11.0 years). The baseline characteristics of the participants are presented in Table 1.

**Table 1:** Baseline characteristics of the participants Age, BMI, and work hours are presented as mean ± standard deviation. Working hours are expressed in hours per day. n, number; BMI, body mass index

Variable	Pain+ (n=15)	Pain– (n=13)
Age	43.8 ± 13.3	37.9 ± 11.0
Sex (men/women)	13/2	10/2
BMI	22.0 ± 1.6	22.4 ± 2.3
Work hours	8.4 ± 0.6	8.8 ± 0.9

Autonomic indices

HRV results are summarized in Table 2. No statistically significant between-group differences were observed for TP or the LF/HF (two-tailed p = 0.139 and 0.525, respectively). HF, an index of parasympathetic activity, was lower in the CMP+ group. While this difference did not reach significance using a two-tailed test (t(26) = −1.74, p = 0.093), it met the threshold for significance under the pre-specified one-tailed test (p = 0.047), with a medium-to-large effect size (Cohen’s d = 0.66).

**Table 2:** Heart rate variability results. n, number; TP, total power; HF, high-frequency power; LF, low-frequency to high-frequency ratio

Variable	Pain+ (n=15)	Pain– (n=13)	t	df	p-value (two-tailed)	Cohen`s d
TP	6.31 ± 0.39	6.71 ± 0.91	-1.53	26	0.139	0.58
HF	4.49 ± 1.06	5.25 ± 1.25	-1.74	26	0.093	0.66
LF/HF	1.09 ± 0.29	1.03 ± 0.21	0.65	26	0.525	0.24

Presenteeism

Given the non-normal distribution of SPQ scores (Shapiro-Wilk p < 0.05), group comparisons were conducted using the Mann-Whitney U test. The CMP+ group reported significantly lower work productivity, with a median SPQ score of 80.0% (interquartile range (IQR): 75-88) compared with 92.0% (IQR: 86-97) in the CMP- group (U = 140.5, z = −2.03, two-tailed p = 0.042), corresponding to a medium effect size (r = 0.38).

Mood

Table 3 presents the four TDMS - vitality, stability, pleasure, and arousal - stratified by the presence or absence of CMP. All four TDMS subscores were normally distributed and compared using independent-samples t-tests. No significant differences were found between groups for vitality (t(26) = −0.50, p = 0.620), stability (t(26) = −0.31, p = 0.760), pleasure (t(26) = −0.47, p = 0.644), and arousal (t(26) = −0.17, p = 0.864). Effect sizes across all mood indices were small (Cohen’s d = −0.19 to −0.07), suggesting negligible differences in momentary emotional states.

**Table 3:** TDMS-ST results. n, number; TDMS-ST, short form of the Two-Dimensional Mood Scale

Variable	Pain+ (n=15)	Pain– (n=13)	t	df	p-value (two-tailed)	Cohen`s d
Vitality	2.13 ± 3.02	2.69 ± 2.84	-0.50	26	0.62	0.19
Stability	3.33 ± 3.22	3.69 ± 2.87	-0.31	26	0.76	0.12
Pleasure	5.47 ± 5.74	6.38 ± 4.43	-0.47	26	0.644	0.18
Arousal	-1.20 ± 2.46	-1.00 ± 3.61	-0.17	26	0.864	0.07

## Discussion

To our knowledge, this pilot study is the first to simultaneously assess autonomic nervous system activity, work productivity, and momentary mood during actual working hours in desk-based employees with CMP. Three key findings emerged. First, parasympathetic activity, as indexed by HF, was reduced in the CMP group, aligning with prior laboratory and clinical evidence of vagal withdrawal among individuals experiencing chronic pain [19]. Second, workers with CMP reported significantly greater productivity loss, as measured by the single-item SPQ, corresponding to a medium effect size. Third, no statistically significant differences were observed between groups across the four TDMS subscales.

Autonomic and productivity implications

The observed reduction in HF suggests diminished vagal modulation and heightened physiological stress. Experimental studies have demonstrated that nociceptive input activates ascending pain pathways and disrupts autonomic balance, contributing to increased sympathetic tone, pro-inflammatory cytokine release, and central pain sensitization [20]. The present findings extend this evidence by demonstrating that such autonomic dysregulation is detectable in real-world occupational settings, independent of laboratory-induced stressors. Concurrently, the reduction in SPQ scores highlights the tangible functional consequences of this physiological strain. A 12-point decrease in SPQ corresponds to an approximate 12% reduction in productivity, which, when extrapolated to an annual salary of ¥5 million, represents a potential economic loss of ¥0.6 million (approximately US$4,000) per affected employee per year. Together, HF and SPQ appear to index distinct but interrelated dimensions of CMP-related burden: biological stress reactivity and behavioral performance, respectively.

Why did mood not differ?

The lack of significant differences in TDMS mood scores may be attributable to several methodological and conceptual factors.

Temporal Resolution

The TDMS assesses momentary affect, whereas the emotional toll of chronic pain is often cumulative and diffuse. A single afternoon measurement may fail to capture such long-term psychological burden.

Measurement Timing

Limiting data collection to the 15:00-16:00 window may have introduced confounding influences related to circadian rhythms or task-related stressors (e.g., postprandial fatigue, approaching work deadlines).

Subjectivity of Self-Report

Mood assessments are inherently susceptible to social desirability and symptom minimization biases, particularly in workplace settings.

Collectively, these speculative factors may have contributed to a “silent gap,” wherein participants reported neutral mood states despite exhibiting both autonomic dysregulation and impaired work productivity. Future studies employing repeated mood assessments, objective workload metrics, and larger samples are needed to empirically test these proposed mechanisms. These findings suggest that reliance on self-reported mood alone may substantially underestimate the burden of CMP in occupational contexts.

Strengths and limitations

This study offers several methodological strengths, including real-time physiological monitoring during actual work activities, integration of autonomic, psychological, and productivity-related metrics, and the use of a validated single-item presenteeism measure that minimizes respondent burden. However, several limitations warrant consideration. First, the small sample size (n = 28) limits statistical power and results in wide confidence intervals. Second, the cross-sectional design precludes causal inference. Third, recruitment from a single organization and occupational role restricts the generalizability of findings. Fourth, reliance on self-report measures for pain, mood, and productivity introduces the potential for reporting biases and lacks corroboration by objective behavioral data. Fifth, baseline characteristics were limited to age, sex, BMI, and daily working hours; variables such as job tenure and pain duration were not obtained. This restricted dataset precluded thorough confounder adjustment and limited the generalizability of our findings. Sixth, because only a limited set of baseline variables (age, sex, BMI, and working hours) was collected, multivariable adjustment for potential confounders could not be performed; future studies with larger samples should incorporate comprehensive demographic and occupational data to enable such analyses.

Future directions

Larger, multi-site longitudinal studies are needed to replicate these findings and examine temporal relationships, specifically whether chronic pain attenuates vagal activity or whether diminished vagal tone predisposes individuals to pain. Randomized controlled trials investigating the efficacy of ergonomic adjustments, physical activity, or biofeedback interventions in restoring HF and improving SPQ scores are also warranted. Additionally, combining HRV assessment with objective digital indicators, such as real-time computer analytics or wearable actigraphy, may facilitate the development of a comprehensive digital phenotype for monitoring pain-related declines in workplace productivity.

## Conclusions

This pilot cross-sectional study of desk-based employees in an IT company demonstrates that CMP is associated with concurrent reductions in parasympathetic activity, as measured by HF-HRV, and decreased work productivity, as measured by the single-item SPQ, during regular working hours. These findings underscore the utility of combining brief HRV monitoring with a single-item productivity assessment as a feasible approach for workplace pain screening and monitoring. To establish causality and inform evidence-based interventions, larger multi-site longitudinal and randomized controlled studies are warranted.
